# NMDA receptor gene variations as modifiers in Huntington disease: a replication study

**DOI:** 10.1371/currents.RRN1247

**Published:** 2011-10-04

**Authors:** Carsten Saft, Jörg T. Epplen, Stefan Wieczorek, G. Bernhard Landwehrmeyer, Raymund A.C. Roos, Justo Garcia de Yebenes, Matthias Dose, Sarah J Tabrizi, David Craufurd, Investigators of the European Huntington's Disease Network, K Barth, M Bascuñana Garde, R Bos, D Ecker, OJ Handley, N Heinonen, C Held, M Laurà, A Martínez Descals, T Mestre, D Monza, J Naji, M Orth, H Padieu, S Pro Koivisto, A Rialland, P Sasinková, P Trigo Cubillo, M van Walsem, M-N Witjes-Ané, D Zielonka, Raphael M. Bonelli, Brigitte Herranhof, Anna Hödl, Hans-Peter Kapfhammer, Michael Koppitz, Markus Magnet, Daniela Otti, Annamaria Painold, Karin Reisinge, Florian Brugger, Caroline Hepperger, Anna Hotter, Philipp Mahlknecht, Michael Nocker, Klaus Seppi, Gregor Wenning, Pascale Ribaï, Christine Verellen-Dumoulin, Jiří Klempíř, Martin Kucharik, Jan Roth, Lis Hasholt, Lena E. Hjermind, Oda Jakobsen, Jørgen E Nielsen, Anne Nørremølle, Sven Asger Sørensen, Jette Stokholm, Heli Hiivola, Kirsti Martikainen, Katri Tuuha, Christoph Michael Kosinski, Daniela Probst, Christian Sass, Johannes Schiefer, Christiane Schlangen, Cornelius J. Werner, Herwig Lange, Matthias Löhle, Alexander Storch, Anett Wolz, Martin Wolz, Johann Lambeck, Birgit Zucker, Alexander Münchau, Lars Stubbe, Simone Zittel, Walburgis Heinicke, Bernhard Longinus, Alexander Peinemann, Michael Städtler, Adolf Weindl, Stefan Bohlen, Herwig Lange, Ralf Reilmann, Antonie Beister, Matthias Dose, Kathrin Hammer, Gabriele Leythaeuser, Ralf Marquard, Tina Raab, Caroline Schrenk, Michele Schuierer, Alexandra Wiedemann, Antonie Beister, Matthias Dose, Kathrin Hammer, Gabriele Leythaeuser, Ralf Marquard, Tina Raab, Caroline Schrenk, Michele Schuierer, Alexandra Wiedemann, Daniel Ecker, Carolin Eschenbach, Bernhard Landwehrmeyer, Franziska Lezius, Michael Orth, Sonja Trautmann, Claudia Cormio, Olimpia Difruscolo, Marina de Tommaso, Vittorio Sciruicchio, Claudia Serpino, Elisabetta Bertini, Claudia Mechi, Marco Paganini, Sivia Piacentini, Maria Romoli, Sandro Sorbi, Giovanni Abbruzzese, Emilio Di Maria, Monica Bandettini di Poggio Giovanna Ferrandes, Paola Mandich, Roberta Marchese, Alberto Albanese, Stefano Di Donato, Caterina Mariotti, Paola Soliveri, Cinzia Gellera, Daniela Monza, Chiara Tomasello, Lorenzo Nanetti, Di Maio Luigi, Giuseppe De Michele, Carlo Rinaldi, Cinzia Russo, Elena Salvatore, Tecla Tucci, Ferdinando Squitieri, Tiziana Martino, Sara Orobello, Silvia Alberti, Francesca De Gregorio, Valentina Codella, Nunzia De Nicola, Vittorio Maglione, Anna Rita Bentivoglio, Alfonso Fasano, Marina Frontali, Arianna Guidubaldi, Tamara Ialongo, Gioia Jacopini, Giovanna Loria, Carla Piano, Silvia Romano, Francesco Soleti, Maria Spadaro, Paola Zinzi, Arvid Heiberg, Marleen R van Walsem, Kathrine Bjørgo, Madelein Fannemel, Per Gørvell, Lars Retterstøl, Inga Bjørnevoll, Sigrid Botne Sando, Jaroslaw Slawek, Witold Soltan, Emilia Sitek, Magdalena Boczarska-Jedynak, Barbara Jasinska-Myga, Gregorz Opala, Andrzej Szczudlik, Monika Rudzińska, Magdalena Wójcik, Krzysztof Banaszkiewicz, Malgorzata Krawczyk, Daniel Zielonka, Jerzy Marcinkowski, Anna Ciesielska, Justyna Sempołowicz, Anna Bryl, Aneta Klimberg, Piotr Janik, Anna Kalbarczyk, Hubert Kwiecinski, Zygmunt Jamrozik, Grzegorz Witkowski, Danuta Ryglewicz, Jakub Antczak, Maria Rakowicz, Katarzyna Jachinska, Elzbieta Zdzienicka, Przemyslaw Richter, Jacek Zaremba, Miguel Coelho, Joaquim J Ferreira, Tiago Mestre, Mário M Rosa, Anabela Valadas, Miguel Gago, Carolina Garrett, Maria Rosalia Guerra, Francisco J Barrero, Blas Morales, José Luis López-Sendón Moreno, Esther Cubo, Natividad Mariscal, Jesús Sánchez, Rocío García, Clara Villanueva, Purificacion Pin Quiroga, Mónica Bascuñana, Patricia Trigo Cubillo, Marta Fatàas, José Luis López Moreno, Guillermo García Ribas, Christine Schwarz, Justo García de Yébenes, María José Saiz Artiga, Asunción Martínez-Descals, Pedro J García Ruíz, Vicenta Sánchez, Lorenza Fortuna Alcaraz, María Fuensanta Noguera Perea, María Martirio Antequera Torres, Laura Vivancos Moreau, Ana Rojo Sebastian, Miquel Aguilar Barbera, Dolors Badenes Guia, Laura Casas Hernanz, Gemma Tome Carruesco, Esther Suarez San Martin, Judit López Catena, Jordi Bas, Matilde Calopa, Núria Busquets, Penelope Navas Arques, Aranzazú Gorospe, Inés Legarda, María José Torres Rodríguez, Barbara Vives, Fátima Carrillo, Pablo Mir, María José Lama Suarez, Ghada Loutfi, Eva-Lena Stattin, Laila Westman, Birgitta Wikström, Sven Pålhagen, Elisabeth Björnsson, Jean-Marc Burgunder, Irene Romero, Michael Schüpbach, Sabine Weber Zaugg, Monique S.E. van Hout, Jeroen P.P. van Vugt, A. Marit de Weert, J.J.W. Bolwijn, M. Dekker, K.L. Leenders, J.C.H. van Oostrom, Reineke Bos, Eve M. Dumas, Caroline K. Jurgens, Simon J. A. van den Bogaard, Raymund A.C. Roos, Marie-Noëlle Witjes-Ané, Berry Kremer, C.C.P. Verstappen, Jenny de Souza, Hugh Rickards, Jan Wright, Roger A. Barker, Kate Fisher, Anna Olivia Goyder Goodman, Susan Hill, Ann Kershaw, Sarah Mason, Nicole Paterson, Lucy Raymond, Johnathan Bisson, Monica Busse, Lynda Ellison-Rose, Olivia Handley, Sarah Hunt, Jenny Naji, Kathleen Price, Anne Rosser, Stephen Dunnett, Maureen Edwards, Paul A. De Sousa, Teresa Hughes, Marie McGill, Pauline Pearson, Mary Porteous, Paul Smith, Adam Zeman, Nicol Lambord, Julia Rankin, Liz Burrows, Amy Fletcher, Fiona Laver, Mark Silva, Aileen Thomson, Thomasin Andrews, Andrew Dougherty, Fred Kavalier, Charlotte Golding, Alison Lashwood, Dene Robertson, Deborah Ruddy, Anna Whaite, Michael Patton, Maria Patterson, Colin Bourne, Carole Clayton, Heather Dipple, Jackie Clapton, Janet Grant, Diana Gross, Caroline Hallam, Julia Middleton, Ann Murch, Dawn Patino, Thomasin Andrews, Stefania Bruno, Elvina Chu, Karen Doherty, Nayana Lahiri, Marianne Novak, Aakta Patel, Sarah Tabrizi, Rachel Taylor, Thomas Warner, Edward Wild, Natalie Arran, David Craufurd, Ruth Fullam, Liz Howard, Susan Huson, Lucy Partington-Jones, Nichola Verstraelen, Julie Snowden, Andrea Sollom, Cheryl Stopford, Jennifer Thompson, Leann Westmoreland, Andrea H Nemeth, Gill Siuda, Oliver Bandmann, Alyson Bradbury, Kay Fillingham, Isabella Foustanos, Katherine Tidswell, Oliver Quarrell, Larissa Arning

**Affiliations:** Language coordinator; Language coordinator; Language coordinator; Language coordinator; Language coordinator; Language coordinator; Language coordinator; Language coordinator; Language coordinator; Language coordinator; Language coordinator; Language coordinator; Language coordinator; Language coordinator; Language coordinator; Language coordinator; Language coordinator; Language coordinator; Language coordinator; Language coordinator; Language coordinator; LKH Graz, Abteilung für Psychiatrie, Austria; LKH Graz, Abteilung für Psychiatrie, Austria; LKH Graz, Abteilung für Psychiatrie, Austria; LKH Graz, Abteilung für Psychiatrie, Austria; LKH Graz, Abteilung für Psychiatrie, Austria; LKH Graz, Abteilung für Psychiatrie, Austria; LKH Graz, Abteilung für Psychiatrie, Austria; LKH Graz, Abteilung für Psychiatrie, Austria; LKH Graz, Abteilung für Psychiatrie, Austria; Universitätsklinik für Neurologie, Innsbruck, Austria; Universitätsklinik für Neurologie, Innsbruck, Austria; Universitätsklinik für Neurologie, Innsbruck, Austria; Universitätsklinik für Neurologie, Innsbruck, Austria; Universitätsklinik für Neurologie, Innsbruck, Austria; Universitätsklinik für Neurologie, Innsbruck, Austria; Universitätsklinik für Neurologie, Innsbruck, Austria; Institut de Pathologie et de Génétiqu, C; Institut de Pathologie et de Génétiqu, Charleroi, Belgiu; Centrum extrapyramidových onemocnění, Prague, Czech Republic; Centrum extrapyramidových onemocnění, Prague, Czech Republic; Centrum extrapyramidových onemocnění, Prague, Czech Republic; Hukommelsesklinikken, Rigshospitalet, Copehnhagen, Denmark; Hukommelsesklinikken, Rigshospitalet, Copehnhagen, Denmark; Hukommelsesklinikken, Rigshospitalet, Copehnhagen, Denmark; Hukommelsesklinikken, Rigshospitalet, Copehnhagen, Denmark; Hukommelsesklinikken, Rigshospitalet, Copehnhagen, Denmark; Hukommelsesklinikken, Rigshospitalet, Copehnhagen, Denmark; Hukommelsesklinikken, Rigshospitalet, Copehnhagen, Denmark; Rehabilitation Centre Suvituuli, Turku-Suvituuli, Finland; Rehabilitation Centre Suvituuli, Turku-Suvituuli, Finland; Rehabilitation Centre Suvituuli, Turku-Suvituuli, Finland; Universitätsklinikum Aachen, Neurologische Klinik, Germany; Universitätsklinikum Aachen, Neurologische Klinik, Germany; Universitätsklinikum Aachen, Neurologische Klinik, Germany; Universitätsklinikum Aachen, Neurologische Klinik, Germany; Universitätsklinikum Aachen, Neurologische Klinik, Germany; Universitätsklinikum Aachen, Neurologische Klinik, Germany; Reha Zentrum in Dinslaken im Gesundheitszentrums Lang, Dinslake, Germany; Universitätsklinikum Carl Gustav Carus an der Technischen Universität Dresden, Klinik und Poliklinik für Neurologie, Dresden, Germany; Universitätsklinikum Carl Gustav Carus an der Technischen Universität Dresden, Klinik und Poliklinik für Neurologie, Dresden, Germany; Universitätsklinikum Carl Gustav Carus an der Technischen Universität Dresden, Klinik und Poliklinik für Neurologie, Dresden, Germany; Universitätsklinikum Carl Gustav Carus an der Technischen Universität Dresden, Klinik und Poliklinik für Neurologie, Dresden, Germany; Universitätsklinik Freiburg, Neurologie, Freiburg, Germany; Universitätsklinik Freiburg, Neurologie, Freiburg, Germany; Universitätsklinikum Hamburg-Eppendorf, Klinik und Poliklinik für Neurologie, Hamburg, Germany; Universitätsklinikum Hamburg-Eppendorf, Klinik und Poliklinik für Neurologie, Hamburg, Germany; Universitätsklinikum Hamburg-Eppendorf, Klinik und Poliklinik für Neurologie, Hamburg, Germany; Psychatrium Heiligenhafen, Germany; Klinik für Psychiatrie und Psychotherapie Marburg-Süd, Germany; Huntington-Ambulanz im Neuro-Kopfzentrum - Klinikum rechts der Isar der Neurologischen Klinik und Poliklinik der Technischen Universität München, Germany; Huntington-Ambulanz im Neuro-Kopfzentrum - Klinikum rechts der Isar der Neurologischen Klinik und Poliklinik der Technischen Universität München, Germany; Huntington-Ambulanz im Neuro-Kopfzentrum - Klinikum rechts der Isar der Neurologischen Klinik und Poliklinik der Technischen Universität München, Germany; Universitätsklinikum Münster, Klinik und Poliklinik für Neurologie, Münster, Germany; Universitätsklinikum Münster, Klinik und Poliklinik für Neurologie, Münster, Germany; Universitätsklinikum Münster, Klinik und Poliklinik für Neurologie, Münster, Germany; Isar-Amper-Klinikum - Klinik Taufkirchen (Vils), Germany; Isar-Amper-Klinikum - Klinik Taufkirchen (Vils), Germany; Isar-Amper-Klinikum - Klinik Taufkirchen (Vils), Germany; Isar-Amper-Klinikum - Klinik Taufkirchen (Vils), Germany; Isar-Amper-Klinikum - Klinik Taufkirchen (Vils), Germany; Isar-Amper-Klinikum - Klinik Taufkirchen (Vils), Germany; Isar-Amper-Klinikum - Klinik Taufkirchen (Vils), Germany; Isar-Amper-Klinikum - Klinik Taufkirchen (Vils), Germany; Isar-Amper-Klinikum - Klinik Taufkirchen (Vils), Germany; Taufkirchen, Germany; Taufkirchen, Germany; Taufkirchen, Germany; Taufkirchen, Germany; Taufkirchen, Germany; Taufkirchen, Germany; Taufkirchen, Germany; Taufkirchen, Germany; Taufkirchen, Germany; Universitätsklinikum Ulm, Neurologie, Ulm, Germany; Universitätsklinikum Ulm, Neurologie, Ulm, Germany; Universitätsklinikum Ulm, Neurologie, Ulm, Germany; Universitätsklinikum Ulm, Neurologie, Ulm, Germany; Universitätsklinikum Ulm, Neurologie, Ulm, Germany; Universitätsklinikum Ulm, Neurologie, Ulm, Germany; Dipartimento di Scienze Neurologiche e Psichiatriche Universita' di Bari, Italy; Dipartimento di Scienze Neurologiche e Psichiatriche Universita' di Bari, Italy; Dipartimento di Scienze Neurologiche e Psichiatriche Universita' di Bari, Italy; Dipartimento di Scienze Neurologiche e Psichiatriche Universita' di Bari, Italy; Dipartimento di Scienze Neurologiche e Psichiatriche Universita' di Bari, Italy; Neurologia I- Unita' di Neurogenetica Departimento di Neurologia e Psichiatria, Universita' di Firenze, Italy; Neurologia I- Unita' di Neurogenetica Departimento di Neurologia e Psichiatria, Universita' di Firenze, Italy; Neurologia I- Unita' di Neurogenetica Departimento di Neurologia e Psichiatria, Universita' di Firenze, Italy; Neurologia I- Unita' di Neurogenetica Departimento di Neurologia e Psichiatria, Universita' di Firenze, Italy; Neurologia I- Unita' di Neurogenetica Departimento di Neurologia e Psichiatria, Universita' di Firenze, Italy; Neurologia I- Unita' di Neurogenetica Departimento di Neurologia e Psichiatria, Universita' di Firenze, Italy; Dipartimento di Neuroscienze, Oftalmologia e Genetica (DiNOG), Università di Genova, Italy; Dipartimento di Neuroscienze, Oftalmologia e Genetica (DiNOG), Università di Genova, Italy; Dipartimento di Neuroscienze, Oftalmologia e Genetica (DiNOG), Università di Genova, Italy; Dipartimento di Neuroscienze, Oftalmologia e Genetica (DiNOG), Università di Genova, Italy; Dipartimento di Neuroscienze, Oftalmologia e Genetica (DiNOG), Università di Genova, Italy; Fondazione IRCCS Istituto Neurologico C. Besta, Milan, Italy; Fondazione IRCCS Istituto Neurologico C. Besta, Milan, Italy; Fondazione IRCCS Istituto Neurologico C. Besta, Milan, Italy; Fondazione IRCCS Istituto Neurologico C. Besta, Milan, Italy; Fondazione IRCCS Istituto Neurologico C. Besta, Milan, Italy; Fondazione IRCCS Istituto Neurologico C. Besta, Milan, Italy; Fondazione IRCCS Istituto Neurologico C. Besta, Milan, Italy; Fondazione IRCCS Istituto Neurologico C. Besta, Milan, Italy; Azienda Ospedaliera Universitaria Federico II - Dipartimento di Scienze Neurologiche, Naples, Italy; Azienda Ospedaliera Universitaria Federico II - Dipartimento di Scienze Neurologiche, Naples, Italy; Azienda Ospedaliera Universitaria Federico II - Dipartimento di Scienze Neurologiche, Naples, Italy; Azienda Ospedaliera Universitaria Federico II - Dipartimento di Scienze Neurologiche, Naples, Italy; Azienda Ospedaliera Universitaria Federico II - Dipartimento di Scienze Neurologiche, Naples, Italy; Azienda Ospedaliera Universitaria Federico II - Dipartimento di Scienze Neurologiche, Naples, Italy; Neurogenetics Unit – IRCCS Neuromed, Pozzilli , Italy; Neurogenetics Unit – IRCCS Neuromed, Pozzilli , Italy; Neurogenetics Unit – IRCCS Neuromed, Pozzilli , Italy; Neurogenetics Unit – IRCCS Neuromed, Pozzilli , Italy; Neurogenetics Unit – IRCCS Neuromed, Pozzilli , Italy; Neurogenetics Unit – IRCCS Neuromed, Pozzilli , Italy; Neurogenetics Unit – IRCCS Neuromed, Pozzilli , Italy; Neurogenetics Unit – IRCCS Neuromed, Pozzilli , Italy; Istituto di Neurobiologia e Medicina Molecolare CNR/ Istituto di Neurologia, Dipartimento di Neuroscienze/ CNR Istituto di Scienze e Tecnologie della Cognizione, Rome, Italy; Istituto di Neurobiologia e Medicina Molecolare CNR/ Istituto di Neurologia, Dipartimento di Neuroscienze/ CNR Istituto di Scienze e Tecnologie della Cognizione, Rome, Italy; Istituto di Neurobiologia e Medicina Molecolare CNR/ Istituto di Neurologia, Dipartimento di Neuroscienze/ CNR Istituto di Scienze e Tecnologie della Cognizione, Rome, Italy; Istituto di Neurobiologia e Medicina Molecolare CNR/ Istituto di Neurologia, Dipartimento di Neuroscienze/ CNR Istituto di Scienze e Tecnologie della Cognizione, Rome, Italy; Istituto di Neurobiologia e Medicina Molecolare CNR/ Istituto di Neurologia, Dipartimento di Neuroscienze/ CNR Istituto di Scienze e Tecnologie della Cognizione, Rome, Italy; Istituto di Neurobiologia e Medicina Molecolare CNR/ Istituto di Neurologia, Dipartimento di Neuroscienze/ CNR Istituto di Scienze e Tecnologie della Cognizione, Rome, Italy; Istituto di Neurobiologia e Medicina Molecolare CNR/ Istituto di Neurologia, Dipartimento di Neuroscienze/ CNR Istituto di Scienze e Tecnologie della Cognizione, Rome, Italy; Istituto di Neurobiologia e Medicina Molecolare CNR/ Istituto di Neurologia, Dipartimento di Neuroscienze/ CNR Istituto di Scienze e Tecnologie della Cognizione, Rome, Italy; Istituto di Neurobiologia e Medicina Molecolare CNR/ Istituto di Neurologia, Dipartimento di Neuroscienze/ CNR Istituto di Scienze e Tecnologie della Cognizione, Rome, Italy; Istituto di Neurobiologia e Medicina Molecolare CNR/ Istituto di Neurologia, Dipartimento di Neuroscienze/ CNR Istituto di Scienze e Tecnologie della Cognizione, Rome, Italy; Istituto di Neurobiologia e Medicina Molecolare CNR/ Istituto di Neurologia, Dipartimento di Neuroscienze/ CNR Istituto di Scienze e Tecnologie della Cognizione, Rome, Italy; Istituto di Neurobiologia e Medicina Molecolare CNR/ Istituto di Neurologia, Dipartimento di Neuroscienze/ CNR Istituto di Scienze e Tecnologie della Cognizione, Rome, Italy; Rikshospitalet, Dept. of Medical Genetics, Oslo-RH, Norway; Rikshospitalet, Dept. of Medical Genetics, Oslo-RH, Norway; Ullevål University Hospital, Oslo, Norway; Ullevål University Hospital, Oslo, Norway; Ullevål University Hospital, Oslo, Norway; Ullevål University Hospital, Oslo, Norway; St. Olavs Hospital, Trondheim , Norway; St. Olavs Hospital, Trondheim , Norway; Specialistic Hospital, Gdansk Zaspa, Gdansk, Poland; Specialistic Hospital, Gdansk Zaspa, Gdansk, Poland; Specialistic Hospital, Gdansk Zaspa, Gdansk, Poland; Silesian Medical University Katowice, Poland; Silesian Medical University Katowice, Poland; Silesian Medical University Katowice, Poland; Krakowska Akademia Neurologii, Krakow, Poland; Krakowska Akademia Neurologii, Krakow, Poland; Krakowska Akademia Neurologii, Krakow, Poland; Krakowska Akademia Neurologii, Krakow, Poland; Krakowska Akademia Neurologii, Krakow, Poland; Medical University of Poznań, Poland; Medical University of Poznań, Poland; Medical University of Poznań, Poland; Medical University of Poznań, Poland; Medical University of Poznań, Poland; Medical University of Poznań, Poland; Medical University of Warsaw, Neurology, Warsaw-MU, Poland; Medical University of Warsaw, Neurology, Warsaw-MU, Poland; Medical University of Warsaw, Neurology, Warsaw-MU, Poland; Medical University of Warsaw, Neurology, Warsaw-MU, Poland; Institute of Psychiatry and Neurology Dep. of Genetics, Dep. of Neurology, Warsaw-IPiN, Poland; Institute of Psychiatry and Neurology Dep. of Genetics, Dep. of Neurology, Warsaw-IPiN, Poland; Institute of Psychiatry and Neurology Dep. of Genetics, Dep. of Neurology, Warsaw-IPiN, Poland; Institute of Psychiatry and Neurology Dep. of Genetics, Dep. of Neurology, Warsaw-IPiN, Poland; Institute of Psychiatry and Neurology Dep. of Genetics, Dep. of Neurology, Warsaw-IPiN, Poland; Institute of Psychiatry and Neurology Dep. of Genetics, Dep. of Neurology, Warsaw-IPiN, Poland; Institute of Psychiatry and Neurology Dep. of Genetics, Dep. of Neurology, Warsaw-IPiN, Poland; Institute of Psychiatry and Neurology Dep. of Genetics, Dep. of Neurology, Warsaw-IPiN, Poland; Neurological Clinical Research Unit, Institute of Molecular Medicine, Lisbon-Santa Maria, Portugal; Neurological Clinical Research Unit, Institute of Molecular Medicine, Lisbon-Santa Maria, Portugal; Neurological Clinical Research Unit, Institute of Molecular Medicine, Lisbon-Santa Maria, Portugal; Neurological Clinical Research Unit, Institute of Molecular Medicine, Lisbon-Santa Maria, Portugal; Neurological Clinical Research Unit, Institute of Molecular Medicine, Lisbon-Santa Maria, Portugal; Hospital São João E.P.E., Porto-São João, Portugal; Hospital São João E.P.E., Porto-São João, Portugal; Hospital São João E.P.E., Porto-São João, Portugal; Hospital Universitario San Cecilio, Neurología, Granada, Spain; Hospital Universitario San Cecilio, Neurología, Granada, Spain; Hospital Universitario Ramón y Cajal, Neurología, Madrid, Spain; Servicio de Neurología Hospital General Yagüe, Burgos, Spain; Servicio de Neurología Hospital General Yagüe, Burgos, Spain; Servicio de Neurología Hospital General Yagüe, Burgos, Spain; Hospital Clínico Universitario San Carlos, Madrid-Clinico, Spain; Hospital Clínico Universitario San Carlos, Madrid-Clinico, Spain; Hospital Clínico Universitario San Carlos, Madrid-Clinico, Spain; Hospital Ramón y Cajal, Neurología, Madrid RYC, Spain; Hospital Ramón y Cajal, Neurología, Madrid RYC, Spain; Hospital Ramón y Cajal, Neurología, Madrid RYC, Spain; Hospital Ramón y Cajal, Neurología, Madrid RYC, Spain; Hospital Ramón y Cajal, Neurología, Madrid RYC, Spain; Hospital Ramón y Cajal, Neurología, Madrid RYC, Spain; Hospital Ramón y Cajal, Neurología, Madrid RYC, Spain; Madrid-Fundación Jiménez Díaz, Madrid FJD, Spain; Madrid-Fundación Jiménez Díaz, Madrid FJD, Spain; Madrid-Fundación Jiménez Díaz, Madrid FJD, Spain; Madrid-Fundación Jiménez Díaz, Madrid FJD, Spain; Hospital Universitario Virgen de la Arrixaca, Murcia, Spain; Hospital Universitario Virgen de la Arrixaca, Murcia, Spain; Hospital Universitario Virgen de la Arrixaca, Murcia, Spain; Hospital Universitario Virgen de la Arrixaca, Murcia, Spain; Barcelona-Hospital Mútua de Terrassa, Spain; Barcelona-Hospital Mútua de Terrassa, Spain; Barcelona-Hospital Mútua de Terrassa, Spain; Barcelona-Hospital Mútua de Terrassa, Spain; Barcelona-Hospital Mútua de Terrassa, Spain; Barcelona-Hospital Mútua de Terrassa, Spain; Barcelona-Hospital Mútua de Terrassa, Spain; Hospital Universitari de Bellvitge, Barcelona-Bellvitge, Spain; Hospital Universitari de Bellvitge, Barcelona-Bellvitge, Spain; Hospital Universitari de Bellvitge, Barcelona-Bellvitge, Spain; Hospital Son Dureta, Palma, Spain; Hospital Son Dureta, Palma, Spain; Hospital Son Dureta, Palma, Spain; Hospital Son Dureta, Palma, Spain; Hospital Son Dureta, Palma, Spain; Hospital Virgen del Rocío, Sevilla, Spain; Hospital Virgen del Rocío, Sevilla, Spain; Hospital Virgen del Rocío, Sevilla, Spain; Norrlands Universitet Sjukhus, Department of Neurology, Umeå, Sweden; Norrlands Universitet Sjukhus, Department of Neurology, Umeå, Sweden; Norrlands Universitet Sjukhus, Department of Neurology, Umeå, Sweden; Norrlands Universitet Sjukhus, Department of Neurology, Umeå, Sweden; Karolinska-University Hospital, Stockholm, Sweden; Karolinska-University Hospital, Stockholm, Sweden; Neurologische Klinik des Inselspitals, Bern, Switzerland; Zentrum für Bewegungsstörungen, Neurologische Klinik und Poliklinik, Bern,Switzerland; Zentrum für Bewegungsstörungen, Neurologische Klinik und Poliklinik, Bern,Switzerland; Zentrum für Bewegungsstörungen, Neurologische Klinik und Poliklinik, Bern,Switzerland; Medisch Spectrum Twente, Ensched, The Netherlands; Medisch Spectrum Twente, Ensched, The Netherlands; Medisch Spectrum Twente, Ensched, The Netherlands; Polikliniek Neurologie, Groningen, The Netherlands; Polikliniek Neurologie, Groningen, The Netherlands; Polikliniek Neurologie, Groningen, The Netherlands; Polikliniek Neurologie, Groningen, The Netherlands; Leiden University Medical Centre, Leiden, The Netherlands; Leiden University Medical Centre, Leiden, The Netherlands; Leiden University Medical Centre, Leiden, The Netherlands; Leiden University Medical Centre, Leiden, The Netherlands; Leiden University Medical Centre, Leiden, The Netherlands; Leiden University Medical Centre, Leiden, The Netherlands; Universitair Medisch Centrum St. Radboud, Neurology, Nijmegen, The Netherlands; Universitair Medisch Centrum St. Radboud, Neurology, Nijmegen, The Netherlands; The Barberry Centre, Dept of Psychiatry, Birmingham, UK; The Barberry Centre, Dept of Psychiatry, Birmingham, UK; The Barberry Centre, Dept of Psychiatry, Birmingham, UK; Cambridge Centre for Brain Repair, Forvie Site, Cambridge, UK; Cambridge Centre for Brain Repair, Forvie Site, Cambridge, UK; Cambridge Centre for Brain Repair, Forvie Site, Cambridge, UK; Cambridge Centre for Brain Repair, Forvie Site, Cambridge, UK; Cambridge Centre for Brain Repair, Forvie Site, Cambridge, UK; Cambridge Centre for Brain Repair, Forvie Site, Cambridge, UK; Cambridge Centre for Brain Repair, Forvie Site, Cambridge, UK; Cambridge Centre for Brain Repair, Forvie Site, Cambridge, UK; The Institute of Medical Genetics, University Hospital of Wales, Cardiff, UK; The Institute of Medical Genetics, University Hospital of Wales, Cardiff, UK; The Institute of Medical Genetics, University Hospital of Wales, Cardiff, UK; The Institute of Medical Genetics, University Hospital of Wales, Cardiff, UK; The Institute of Medical Genetics, University Hospital of Wales, Cardiff, UK; The Institute of Medical Genetics, University Hospital of Wales, Cardiff, UK; The Institute of Medical Genetics, University Hospital of Wales, Cardiff, UK; The Institute of Medical Genetics, University Hospital of Wales, Cardiff, UK; The Institute of Medical Genetics, University Hospital of Wales, Cardiff, UK; Molecular Medicine Centre, Western General Hospital, Department of Clinical Genetics, Edinburgh, UK; Molecular Medicine Centre, Western General Hospital, Department of Clinical Genetics, Edinburgh, UK; (Scottish Huntington´s Association), Molecular Medicine Centre, Western General Hospital, Department of Clinical Genetics, Edinburgh, UK; Molecular Medicine Centre, Western General Hospital, Department of Clinical Genetics, Edinburgh, UK; Molecular Medicine Centre, Western General Hospital, Department of Clinical Genetics, Edinburgh, UK; Molecular Medicine Centre, Western General Hospital, Department of Clinical Genetics, Edinburgh, UK; (Scottish Huntington´s Association), Molecular Medicine Centre, Western General Hospital, Department of Clinical Genetics, Edinburgh, UK; Molecular Medicine Centre, Western General Hospital, Department of Clinical Genetics, Edinburgh, UK; Heavitree Hospital, Exeter, UK; Heavitree Hospital, Exeter, UK; Department of Neurology Gloucestershire Royal Hospital, Gloucester, UK; Department of Neurology Gloucestershire Royal Hospital, Gloucester, UK; Department of Neurology Gloucestershire Royal Hospital, Gloucester, UK; Department of Neurology Gloucestershire Royal Hospital, Gloucester, UK; Department of Neurology Gloucestershire Royal Hospital, Gloucester, UK; Guy's Hospital, London, UK; Guy's Hospital, London, UK; Guy's Hospital, London, UK; Guy's Hospital, London, UK; Guy's Hospital, London, UK; Guy's Hospital, London, UK; Guy's Hospital, London, UK; Guy's Hospital, London, UK; St. Georges-Hospital, London, UK; St. Georges-Hospital, London, UK; Leicestershire Partnership Trust, Mill Lodge, Leicester, UK; Leicestershire Partnership Trust, Mill Lodge, Leicester, UK; Leicestershire Partnership Trust, Mill Lodge, Leicester, UK; Leicestershire Partnership Trust, Mill Lodge, Leicester, UK; Leicestershire Partnership Trust, Mill Lodge, Leicester, UK; Leicestershire Partnership Trust, Mill Lodge, Leicester, UK; Leicestershire Partnership Trust, Mill Lodge, Leicester, UK; Leicestershire Partnership Trust, Mill Lodge, Leicester, UK; Leicestershire Partnership Trust, Mill Lodge, Leicester, UK; Leicestershire Partnership Trust, Mill Lodge, Leicester, UK; The National Hospital for Neurology and Neurosurgery, London, UK; The National Hospital for Neurology and Neurosurgery, London, UK; The National Hospital for Neurology and Neurosurgery, London, UK; The National Hospital for Neurology and Neurosurgery, London, UK; The National Hospital for Neurology and Neurosurgery, London, UK; The National Hospital for Neurology and Neurosurgery, London, UK; The National Hospital for Neurology and Neurosurgery, London, UK; The National Hospital for Neurology and Neurosurgery, London, UK; The National Hospital for Neurology and Neurosurgery, London, UK; The National Hospital for Neurology and Neurosurgery, London, UK; The National Hospital for Neurology and Neurosurgery, London, UK; Genetic Medicine, University of Manchester, Manchester Academic Health Sciences Centre and Central Manchester University Hospitals NHS Foundation Trust, Manchester, UK; Genetic Medicine, University of Manchester, Manchester Academic Health Sciences Centre and Central Manchester University Hospitals NHS Foundation Trust, Manchester, UK; Genetic Medicine, University of Manchester, Manchester Academic Health Sciences Centre and Central Manchester University Hospitals NHS Foundation Trust, Manchester, UK; Genetic Medicine, University of Manchester, Manchester Academic Health Sciences Centre and Central Manchester University Hospitals NHS Foundation Trust, Manchester, UK; Genetic Medicine, University of Manchester, Manchester Academic Health Sciences Centre and Central Manchester University Hospitals NHS Foundation Trust, Manchester, UK; Genetic Medicine, University of Manchester, Manchester Academic Health Sciences Centre and Central Manchester University Hospitals NHS Foundation Trust, Manchester, UK; (formerly Ritchie), Genetic Medicine, University of Manchester, Manchester Academic Health Sciences Centre and Central Manchester University Hospitals NHS Foundation Trust, Manchester, UK; Genetic Medicine, University of Manchester, Manchester Academic Health Sciences Centre and Central Manchester University Hospitals NHS Foundation Trust, Manchester, UK; Genetic Medicine, University of Manchester, Manchester Academic Health Sciences Centre and Central Manchester University Hospitals NHS Foundation Trust, Manchester, UK; Genetic Medicine, University of Manchester, Manchester Academic Health Sciences Centre and Central Manchester University Hospitals NHS Foundation Trust, Manchester, UK; Genetic Medicine, University of Manchester, Manchester Academic Health Sciences Centre and Central Manchester University Hospitals NHS Foundation Trust, Manchester, UK; Genetic Medicine, University of Manchester, Manchester Academic Health Sciences Centre and Central Manchester University Hospitals NHS Foundation Trust, Manchester, UK; Oxford Radcliffe Hospitals NHS Trust, Oxford, UK; Oxford Radcliffe Hospitals NHS Trust, Oxford, UK; The Royal Hallamshire Hospital, Sheffield, UK; The Royal Hallamshire Hospital, Sheffield, UK; The Royal Hallamshire Hospital, Sheffield, UK; The Royal Hallamshire Hospital, Sheffield, UK; The Royal Hallamshire Hospital, Sheffield, UK; The Royal Hallamshire Hospital, Sheffield, UK; ^*^Department of Neurology, Huntington-Center NRW, Ruhr-University, St. Josef-Hospital, Bochum, Germany; ^†^Department of Human Genetics, Ruhr-University, Bochum, Germany; ^§^Department of Neurology, University of Ulm, Ulm, Germany; ^¶^LUMC, Leiden, The Netherlands; ^#^Department of Neurology, Hospital Ramon y Cajal, Madrid, Spain; ^**^Isar-Amper-Klinikum, Klinik Taufkirchen, Germany; ^††^Department of Neurodegenerative Disease, UCL Institute of Neurology, London, UK and ^‡‡^University of Manchester, Manchester Academic Health Sciences Centre and Central Manchester University Hospitals NHS Foundation Trust, Manchester, UK

## Abstract

Several candidate modifier genes which, in addition to the pathogenic CAG repeat expansion, influence the age at onset (AO) in Huntington disease (HD) have already been described. The aim of this study was to replicate association of variations in the N-methyl D-aspartate receptor subtype genes GRIN2A and GRIN2B in the “REGISTRY” cohort from the European Huntington Disease Network (EHDN). The analyses did replicate the association reported between the GRIN2A rs2650427 variation and AO in the entire cohort. Yet, when subjects were stratified by AO subtypes, we found nominally significant evidence for an association of the GRIN2A rs1969060 variation and the GRIN2B rs1806201 variation. These findings further implicate the N-methyl D-aspartate receptor subtype genes as loci containing variation associated with AO in HD.

## **Introduction**

Huntington disease (HD) is an autosomal dominant neurodegenerative disorder characterised by motor disturbances, cognitive decline, and neuropsychiatric symptoms. It is caused by a CAG repeat expansion (>36 repeats) in exon 1 of the *HTT* gene.^[1]^ The lengths of the expanded CAG tract is inversely related to the age at clinical onset of HD, accounting for more than half of the overall variance in age at onset (AO).^[2]^ Despite this strong correlation, there remains considerable variation of over 40 years in AO in individuals with identical repeat lengths. Several candidate modifier genes of HD have already been described in independent studies.^[3][4][5][6][7][8][9]^ In order to confirm the associations between modifier gene variations and AO, independent replication studies are compulsory. Here, we tested the primary hypothesis of an original study ^[4]^, that variations in the NR2A and NR2B glutamate receptor subunit genes (*GRIN2A*, *GRIN2B*) explain additional variance in AO for HD.

## **Methods**

The study cohort comprised 1,211 individuals of European ancestry with HD collected by the EHDN “REGISTRY” study prior to October 14, 2008. “REGISTRY” is a multi-centre, multi-national observational study which aims to obtain natural history data on a wide spectrum of the European HD population (http://www.euro-hd.net/html/registry).^[10]^ In order to test previously reported HD genetic modifiers in this cohort, HD patients with available data on age, sex, age at symptom onset, mutant CAG repeat size and body mass index (BMI) were included (initial *n* = 1211; * n* = 1069; 529 men and 540 women had a complete data set).

The expanded trinucleotide repeats ranged from 40 to 89 with a mean (± SD) of 45±4.7 CAGs, and AO ranged from 6 to 74 years, with an onset (mean ± SD) of 42 ±11.8 years. AO was defined as the age at which, according to the rater, the first signs of HD appeared. Five hundred and thirty-eight patients first presented with motor disturbances (mean ± SD motor AO = 43.4±11.6 years), 241 with psychiatric problems (mean ± SD psychiatric AO = 39.9±10.8 years), and 112 with cognitive decline (mean ± SD cognitive AO = 38.6±13.1 years). For the remaining patients no specific symptoms were listed (mean ± SD AO = 42.1±11.8 years). Genotyping of three SNPs was conducted as described before.^[4]^

## **Results**

None of the SNPs deviated from Hardy*-*Weinberg Equilibrium (HWE). Considering the earliest AO (*n* = 1,069), we did find evidence of association of the *GRIN2A* SNP rs2650427 (table). The R^2 ^ statistic rose modestly (from 0.634 to 0.635) but significantly (p=0.028) when *GRIN2A* genotypes were added to the regression model. The analysis did not, however, replicate the association reported between the SNP rs1969060 in intron 2 of the *GRIN2A* gene and SNP C2664T (rs1806201) in exon 12 of the *GRIN2B* gene (table); but when dividing the cohort according to the nature of the symptoms presented initially, both the *GRIN2B* C2664T and the *GRIN2A* rs1969060 polymorphisms explained a small but considerable amount of additional variance in residual AO in the respective samples. Inclusion of the *GRIN2B* genotypes in the model for motor AO (*n* = 538) increased the R^2^ statistic from 0.620 to 0.623 (p=0.046) and in the study of 241 patients with psychiatric AO, the R^2^ statistic of the exponential regression rose from 0.515 to 0.523 with the* GRIN2A* rs1969060 genotypes included (p=0.026, table). Interestingly, the association of cognitive AO (*n * = 112) with the *GRIN2A* rs2650427 polymorphism shows the highest nominal significance as compared to the other models in the study (0.770 to 0.775, p=0.014). Yet, the results remain statisticallysignificant when excluding the patients with CAGs over >70 (n=4).   

**Table:** 

Model	Genotypes	CAG mean ± SD	**erliest AO** mean ± SD	R²*	P value
HD CAG 40-89 (*n*=1069)		44.97±4.7	41.87±11.8	0.634	<0.0005
+ *GRIN2B* C2664T (rs1806201)	CC (n=560)	44.94±4.8	42.13±12.1	0.634	0.541
	CT (n=436)	45.02±4.5	41.32±11.3	0.634	0.199
	TT (n=73)	44.86±4.6	43.14±11.9	0.634	0.196
	additive			0.634	0.973
+ *GRIN2A* rs1969060	TT (n=745)	44.83±4.5	42.00±11.9	0.634	0.160
	TC (n=292)	45.24±5.1	41.67±11.5	0.634	0.203
	CC (n=32)	45.75±4.2	40.66±11.5	0.634	0.645
	additive			0.634	0.172
+ *GRIN2A* rs2650427	CC (n=374)	44.99±4.0	41.40±11.9	0.634	0.125
	CT (n=512)	44.94±5.2	41.99±11.9	0.634	0.891
	TT (n=183)	42.51±4.2	43.14±10.9	**0.635**	**0.031**
	additive			**0.635**	**0.028**
Model	Genotypes	CAG mean ± SD	**motor AO** mean ± SD	R²*	P value
HD CAG 40-77 (*n*=538)		44.72±4.0	**43.39±11.6**	0.620	<0.0005
+ *GRIN2B* C2664T (rs1806201)	CC (n=274)	44.52±3.8	44.41±11.8	0.622	0.099
	CT (n=221)	44.96±4.1	41.98±11.1	**0.623**	**0.046**
	TT (n=43)	44.77±4.9	44.07±12.4	0.620	0.560
	additive			0.621	0.296
+ *GRIN2A* rs1969060	TT (n=373)	44.51±3.6	43.79±11.5	0.620	0.824
	TC (n=153)	45.32±4.9	42.19±11.9	0.620	0.934
	CC (n=12)	43.75±2.5	46.25±9.5	0.620	0.658
	additive			0.620	0.744
+ *GRIN2A* rs2650427	CC (n=181)	44.15±3.8	41.99±11.8	0.620	0.362
	CT (n=259)	44.48±4.1	44.00±11.9	0.620	0.792
	TT (n=98)	44.60±4.2	44.34±10.4	0.621	0.143
	additive			0.621	0.158
Model	Genotypes	CAG mean ± SD	**psychiatric AO** mean ± SD	R²*	P value
HD CAG 40-67 (*n*=241)		44.73±4.1	**39.86±10.8**	0.515	<0.0005
+ *GRIN2B* C2664T (rs1806201)	CC (n=139)	44.81±4.4	39.50±11.3	0.513	0.964
	CT (n=90)	44.74±3.8	39.79±10.5	0.514	0.607
	TT (n=12)	43.67±2.1	44.58±9.6	0.517	0.211
	additive			0.514	0.618
+ *GRIN2A* rs1969060	TT (n=172)	44.79±4.1	39.01±10.9	**0.523**	**0.026**
	TC (n=63)	44.01±3.7	42.78±9.8	**0.523**	**0.033**
	CC (n=6)	49.50±6.8	33.50±15.5	0.514	0.649
	additive			**0.522**	**0.037**
+ *GRIN2A* rs2650427	CC (n=83)	44.79±4.0	39.42±11.3	0.514	0.504
	CT (n=120)	44.24±3.8	40.90±10.5	0.514	0.702
	TT (n=38)	43.13±5.1	37.53±10.8	0.514	0.724
	additive			0.514	0.514
Model	Genotypes	CAG mean ± SD	**cognitive AO** mean ± SD	R²*	P value
HD CAG 40-89 (*n*=112)		46.34±7.8	38.60±13.1	0.765	<0.0005
+ *GRIN2B* C2664T (rs1806201)	CC (n=54)	46.76±8.5	37.89±14.2	0.763	0.621
	CT (n=55)	45.74±7.1	39.73±11.9	0.763	0.530
	TT (n=3)	50.00±6.2	30.67±14.0	0.763	0.682
	additive			0.763	0.742
+ *GRIN2A* rs1969060	TT (n=74)	46.24±7.8	38.95±14.1	0.763	0.682
	TC (n=31)	46.74±8.5	37.68±11.4	0.763	0.799
	CC (n=7)	45.71±3.8	39.00±10.4	0.763	0.741
	additive			0.763	0.650
+ *GRIN2A* rs2650427	CC (n=40)	45.55±4.9	37.80±12.4	**0.770**	**0.054**
	CT (n=55)	47.67±10.0	36.87±13.2	0.763	0.742
	TT (n=17)	43.9±3.2	46.06±12.3	**0.772**	**0.033**
	additive			**0.775**	**0.014**

**Table  ** Thevariability in AO attributable to the CAG repeat length was assessed by linear regression using the logarithmically transformed AO as the dependent variable and *GRIN* genotypes as independent variables. *R^2^ illustrates the relative improvement of the regression model, when the genotypes are considered in addition to the CAG repeats.

In order to control the effect of sex-specific associations, we further analysed each combination of genotype with sex, but there was no trend towards significance. Moreover, on average, psychiatric and cognitive symptoms significantly predate clinical motor onset by 3.5 and 4.8 years (p< 0.001), thus confirming that affective and cognitive symptoms could be early manifestations of neuronal dysfunction.

## **Discussion**

Of the three polymorphisms tested, GRIN2A rs2650427 showed the most consistent evidence of replication in the EHDN Registry study sample. This is in accordance with another replication study in the large set of kindreds from Venezuela, where GRIN2A variation also explained a small but considerable amount of additional variance in residual AO.^[5]^

Yet, the interpretation of the association of cognitve AO with the GRIN2A rs2650427 polymorphism should be considered with caution since the sample size of this subgroub (n=112) is too small to provide the statistical power required.

Unfortunately, none of the SNPs associated has been validated functionally and it is most likely that the polymorphisms analysed are not the functional variations, but represent markers in linkage disequilibrium with variations that modify the AO. Although, synonymous SNPs like GRIN2B rs1806201 might be pathogenetically relevant via influencing mRNA splicing, protein stability and structure.

The failure to replicate the sex-specific effect of rs1806201 suggests that the original observation may have been false positive, emphasizing the need for stringent statistical thresholds.  On the other hand, since linkage disequilibrium is not uniform across populations, the mixed ancestry in the EHDN REGISTRY study sample could account for heterogeneous results. Inconsistent results may also occur because of difficulties in exact AO definitions. The data stresses the need for precise phenotyping in order to reduce heterogeneity, and to facilitate the discovery of clinically relevant biological pathways. 

Although the associations replicated explain only a small fraction of the variance of AO, the observed correlations with HD phenotypes demonstrate that GRIN2A and GRIN2B remain promising candidate genes, worth to be studied further in more detail.

## **Acknowledgments**

The authors thank all EHDN Registry Study Group investigators for collecting the data and all participating patients for their time and efforts.   

Correspondence to Dr Larissa Arning, Ruhr-University, Department of Human Genetics, Universitätsstr. 150, MA5/39,  44801 Bochum, Germany, larissa.arning@rub.de   

## **Competing interests**

The authors have declared that no competing interests exist.

## **Funding information**

The European Huntington’s Disease Network is funded by CHDI Foundation, Inc. CS was supported by FoRUM grant K040-09.

## **Ethics approval**

This study was conducted with the approval of the local ethics committee of the different clinical centres.
